# Impacts of the continuous maize cultivation on soil properties in Sainyabuli province, Laos

**DOI:** 10.1038/s41598-020-67830-9

**Published:** 2020-07-08

**Authors:** Kazuhiko Fujisao, Phanthasin Khanthavong, Saythong Oudthachit, Naruo Matsumoto, Koki Homma, Hidetoshi Asai, Tatsuhiko Shiraiwa

**Affiliations:** 1 Graduate School of Agriculture, Kyoto University, Kitashirakawa-Oiwake, Sakyo, Kyoto, Kyoto 606-8502 Japan; 2 National Agriculture and Forest Research Institute, 811, Nongvienkham, Xaythany, Vientiane Capital Lao People’s Democratic Republic; 3 Japan International Research Center for Agricultural Sciences, 1-1 Ohwashi, Tsukuba, Ibaraki 305-8686 Japan; 4 Graduate School of Agricultural Science, Tohoku University, Aramaki Aza-Aoba, Aoba, Sendai, Miyagi 980-0845 Japan; 5 The United Graduate School of Agricultural Sciences, Kagoshima University, 1-21-24, Korimoto, Kagoshima, 890-0065 Japan

**Keywords:** Agroecology, Sustainability

## Abstract

In tropical mountainous areas, soil degradation and yield decrease have been anticipated due to conversion from shifting to continuous cultivation and the introduction of cash crops. In our previous report, we quantified the decrease in maize yield under continuous cultivation in farmers’ fields in Laos. In this report, we focused on soil nutritional conditions under continuous cultivation in the farmers’ fields. For the purpose, twelve soil properties were investigated over two years from three sample sites in each of the 40 farmers’ fields with the duration of continuous cultivation varying from 1 to 30 years. Total carbon (TC), total nitrogen (TN), available phosphorus, exchangeable potassium, and exchangeable calcium in the soil decreased with increasing duration of continuous cultivation in the sloped fields. These soil nutrients decreased to around half of the initial content in these 30 years. However, the decreasing rates of TC and TN were negligible in the flat fields. Other soil properties such as clay and exchangeable magnesium were not related to the duration of continuous cultivation in both sloped and flat fields. The reduction in maize yield was mainly explained by TC, but the determination coefficient was only 0.24. Although further analysis is required to quantify the effect of soil nutrients on maize production, the development of integrated soil management would be necessary in the sloped fields for sustainable crop production in the study site.

## Introduction

In Southeast Asia, farmers traditionally practiced shifting cultivation in mountainous areas, but they have adopted continuous cultivation in recent years^1,2^. In Laos, maize (*Zea mays* L.) is one of the representative crops produced in converted continuous cultivation. However, maize has been cultivated without fertilizers or any effective soil conservation practices. Thus, we investigated the chronosequential changes in maize productivity in farmers’ fields in Kenthao District in Sainyabuli Province, one of the leading maize production districts in Laos^1^. Our previous report showed that maize production has continued for 30 years, but the yield has decreased gradually. The results also showed that the profitability in 11% of farmers’ fields was already low^1^.

Several studies have reported that the decrease in maize yield is associated with soil degradation under continuous cultivation^4–7^. Lestrelin and Giordano^8^ indicated that soil degradation in upland fields in Laos was induced by a change in the land use system in recent years. Moreover, in tropical climate zones, the soil is easily degraded by high temperatures, which induces decomposition of soil organic matter^9^ and high rainfall intensity, which can accelerate soil erosion, particularly in the rainy season^10^.

Previous studies have also reported that continuous maize cultivation is associated with decreasing soil nutrients due to soil degradation. Dalal and Mayer^11^ reported a decrease in total carbon (TC) during continuous maize cultivation for 70 years in Australia, while Juo et al.^4^ reported a decrease in TC, exchangeable magnesium (Ex-Mg), and exchangeable calcium (Ex-Ca) during continuous maize cultivation for nine years in Nigeria. A reduction in available phosphorus (Av-P) and exchangeable potassium (Ex-K) was also reported in a field investigation during 11 years of maize cultivation in the USA^5^. Similarly, the decreasing trends of Ex-K, Ex-Mg, and Ex-Ca were detected during continuous cultivation for around 80 years in Kenya^7^. The differences observed in decreasing soil nutrients may be associated with climatic and pedological characteristics, rather than local characteristics.

The decreasing rate of soil nutrients is also associated with topological characteristics. The soil at the upper part of the sloped fields rapidly degrades because of erosion, but there is minimal erosion at the lower part because of sedimentation. Pennock et al.^12^ reported a decreasing trend of TC at the upper part of the sloped fields and an increasing trend at the lower part. Additionally, soil erosion increases with increasing slope angle of fields^13–16^. As a majority of maize in Sainyabuli province is grown on steep sloping lands, the effect of soil erosion might be considerable. Therefore, the decreasing rate of soil nutrients with topological characteristics should be analyzed.

The long-term trend of soil conditions was analyzed using the chronosequence method, in which, the soil conditions among discrete fields with various durations under particular cultivation management were compared. This method has been used in several studies^7,17,18^. Nyberg et al.^18^ conducted a field investigation at eight Kenyan sites with various maize cultivation durations to evaluate soil property trends under crop cultivation that had been operating for 120 years. A similar investigation evaluated the trends in soil conditions for 80 years in several farmers’ fields in Kenya^7^. In our previous report, the choronosequence method was used to evaluate the trend of maize yield under continuous cultivation in Kenthao District, Sinyabuli Province^3^.

In this report, we focused on soil nutritional conditions (e.g., TC, total nitrogen (TN), Av-P, exchangeable cation, soil pH, and soil texture) in farmers’ fields in Kenthao District, Sainyabuli Province, Laos. The soil properties were analyzed with the duration of continuous maize cultivation and topo-sequential positions to assess productivity under continuous maize cultivation using the chronosequential method.

## Materials and methods

### Study area

**Figure 1:**
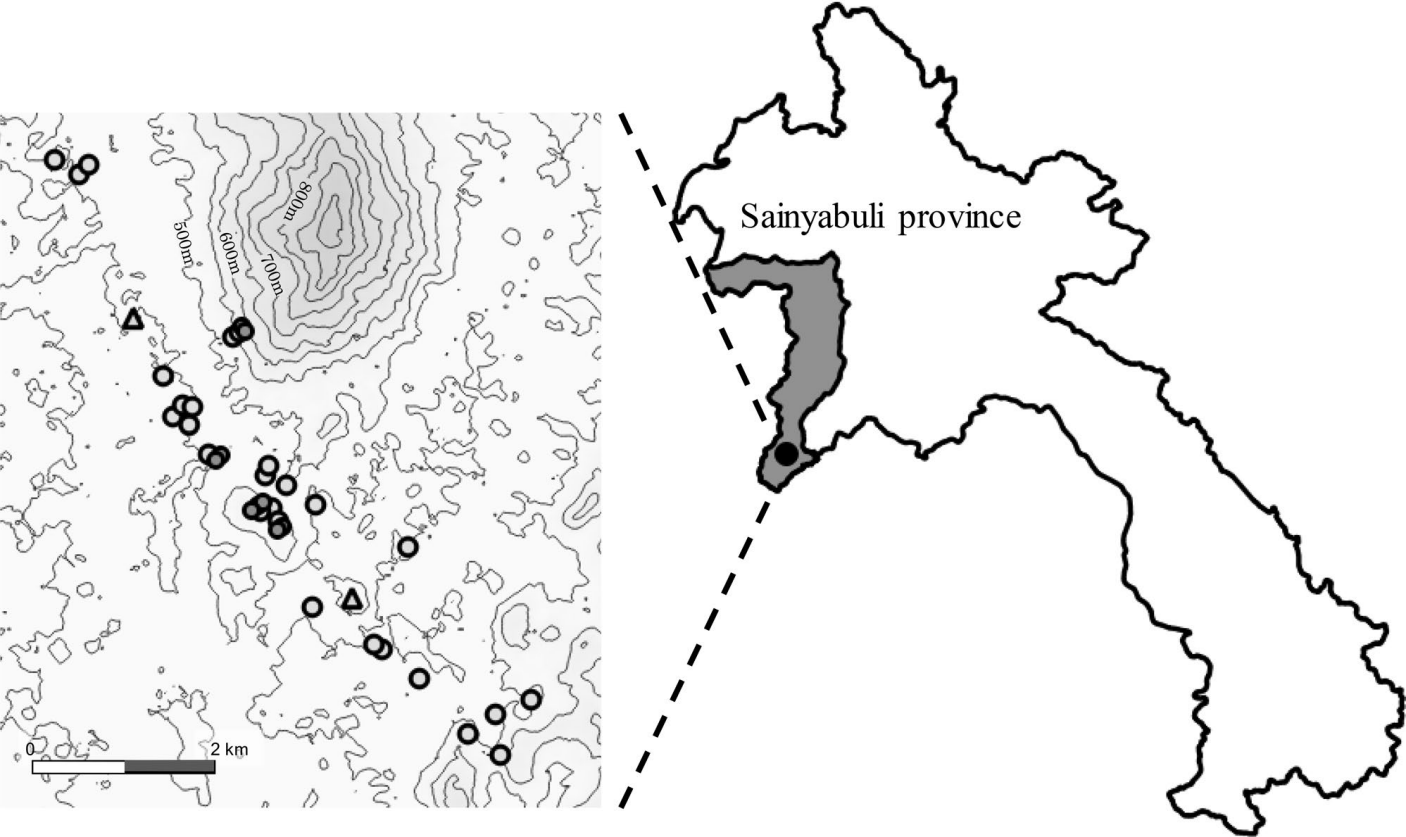
Map of the study area, including the investigation fields and villages in Kenthao District, Sainyabuli Province. The open and closed dircle symbols represent the investigated maize fields and the non-cultivation fields, respectively. The triangles symbols represent the villages.

This research was conducted in the same location as that of a previous study in Kenthao District in Sainyabuli Province^3^ (Fig. 1). The average rainfall and the average temperature in the rainy season in the province are 1,049 mm and 27.5 °C, respectively. Soils in the study area are categorized into Acrisol in the soil map as per the Food and Agriculture Organization taxonomy, and Ultisol in the global soil regions map as per the United States Department of Agriculture taxonomy. Maize cultivation was introduced 30 years ago in Kenthao District. The duration of continuous maize cultivation varied from 1 to 30 years among the fields, depending on the reclamation time when the fields were prepared from the primary or secondary forest. During continuous cropping, the following field management has been conducted in the investigated fields, as reported in our previous study^3^. Only maize was cultivated once in the rainy season, and no crops were cultivated in the dry season. Most of the farmers in our study used hybrid seed varieties from Charoen Pokphand Seeds Co. Ltd. The fields were tilled using tractors with a disk plow between April and May, followed by sowing. After harvesting the grains between September and November, stems and leaves were left in the fields. The farmers did not use any fertilizers or practice fallowing during continuous maize cultivation. This cultivation management was consistent in all of the farmers’ fields investigated in this study.

### Study fields and classification

In 2014 and 2015, 21 and 19 fields were investigated in two villages (Fig. 1), respectively. The field size varied from 0.3 ha to 2 ha and was 0.9 ha on average. Three sample sites were selected to equally distribute the slope with the maximum angle in each field, and the locations of the sample sites were measured with a global positioning system (GPS). To evaluate the effect of topological characteristics on soil properties under continuous cultivation, the sample sites were categorized into four topo-sequential positions: upper, middle, lower, and flat positions. The categorization defined fields into two groups: sloped (i.e., slope angles over 8°) and flat fields. The slope angle of the fields was determined using the positional information of the three sample sites measured with GPS and QGIS using the data from the Advanced Spaceborne Thermal Emission and Reflection Radiometer Global Digital Elevation Model (ASTER GDEM ver. 2, NASA). Subsequently, the sample sites of the sloped fields were categorized into the upper, middle, or lower position per the relative elevation of the three sample sites in each slope field. The three sample sites of each flat field were categorized into flat positions. Nine samples from primary or secondary forest were selected as soil sample references for non-cultivation fields.

### Soil analysis

The soil was collected from the surface (between 0 and − 15 cm) using a scoop from where the canopy formed representative growth at each sample site during maize maturity time from September to October. The following soil analyses were performed after air-drying the soil samples at room temperature and removing gravel from the samples using a 2-mm sieve. TC and TN contents were measured by the combustion method using JM 3,000 (J-Science Lab Co., Ltd.). Av-P was extracted from 1 g of air-dried soil using 20 mL of the extraction liquid by the modified method of Bray No. 2^19^. The extracted Av-P was colored using the vanadate-molybdate reagent and the concentration was determined using a spectrophotometer UV-1600 (Shimadzu Co.). Exchangeable cations were measured using atomic absorption and flame photometry using SPCA-6610 (Shimadzu Co.) after extraction using 1 M ammonium acetate. Soil pH was measured for the solution containing air-dried soil and distilled water in the ratio of 1:2.5 (H_2_O). The gravel content was determined by using 300 g oven-dried soil and a 2 mm sieve. After gravel was removed from the soil samples, clay and silt were separated from the supernatant of the soil suspension using a pipette. After removing clay and silt from the soil suspension, sand sediment was extracted. The weights of clay, silt, and sand were measured after drying them overnight at 105 °C. Following this, the ratio of each soil particle fraction was calculated.

### Maize yield analysis

Yield was quantified during the crop maturity season in September and October. Grain was collected from 3 × 3 m plots where soil samples were collected at each sample site. The grains were dried for 72 h at 70 °C, and then the dry weight was measured. Yield was calculated from the dry weight of the grain seed with a moisture content of 15%.

### Statistical analysis

Analysis of covariance (ANCOVA) was performed to determine the effect of the duration of continuous maize cultivation and the topo-sequential positions on the soil properties. To analyze the relationships between the duration of continuous maize cultivation and soil properties in the sloped fields, linear regression analysis was performed. The initial contents and decreasing rates of soil properties were estimated by the intercepts and slopes in the linear regression line, respectively. ANCOVA was also performed to determine the effect of soil properties and topo-sequential positions on maize yield. R (ver. 3.3.2, R Core Team) was used for all statistical analyses.

## Results

### Characteristics of soil properties

TC ranged between 2.8 and 43.7 g C kg^-1^ in the study fields. Av-P ranged widely between 1.9 and 76.8 mg P_2_O_5_ kg^-1^, but three-quarters of samples were under 8.5 mg P_2_O_5_ kg^-1^. Ex-K also widely ranged between 0.08 and 1.16 cmol_c_ K kg^-1^. The TC, TN, and Ex-K contents were relatively high in non-cultivation fields than in cultivation fields (Table 1). In addition, the contents of TC, TN, Av-P, Ex-K, and Ex-Ca were relatively high in the fields where the duration of continuous cultivation was less than 10 years.

**Table 1:** Decadal difference of the soil properties at each topo-sequential position.

Position	Field type	Cultivation duration (year)	TC (g C kg^−1^)	TN (g N kg^−1^)	Av-P (mg P_2_O_5_ kg^−1^)	Ex-K (cmol_c_ K kg^−1^)	Ex-Mg (cmol_c_ Mg kg^−1^)	Ex-Ca (cmol_c_ Ca kg^−1^)
Non-cultivation fields (n = 9)		0	30 ± 6	2.0 ± 0.3	4.1 ± 1.7	0.60 ± 0.06	21.9 ± 10.6	10 ± 3
Slope fields	Upper position (n = 32)	1–10	31 ± 10	1.9 ± 0.5	13.3 ± 13.0	0.44 ± 0.20	11.0 ± 2.2	30 ± 9
		11–20	17 ± 7	1.3 ± 0.3	4.8 ± 2.9	0.19 ± 0.04	9.9 ± 2.4	21 ± 8
		21–30	18 ± 7	1.3 ± 0.3	4.2 ± 1.4	0.17 ± 0.05	10.8 ± 3.7	25 ± 16
	Middle position (n = 32)	1–10	31 ± 8	2.0 ± 0.4	13.5 ± 11.4	0.52 ± 0.28	10.3 ± 2.9	29 ± 8
		11–20	19 ± 7	1.3 ± 0.3	3.7 ± 1.2	0.26 ± 0.10	11.2 ± 3.6	22 ± 9
		21–30	19 ± 5	1.3 ± 0.3	6.1 ± 6.4	0.25 ± 0.15	10.6 ± 4.6	22 ± 11
	Lower position (n = 32)	1–10	31 ± 9	1.9 ± 0.5	16.7 ± 25.0	0.53 ± 0.31	10.0 ± 2.3	28 ± 6
		11–20	24 ± 7	1.5 ± 0.3	7.3 ± 5.0	0.39 ± 0.17	10.9 ± 3.0	24 ± 7
		21–30	21 ± 5	1.4 ± 0.3	5.2 ± 2.0	0.26 ± 0.13	9.5 ± 4.4	20 ± 10
The flat fields	Flat position (n = 24)	1–10	16 ± 2	1.2 ± 0.1	21.3 ± 13.3	0.26 ± 0.09	4.1 ± 0.4	13 ± 3
		11–20	19 ± 7	1.4 ± 0.3	19.2 ± 19.6	0.34 ± 0.16	8.5 ± 1.8	21 ± 4
		21–30	17 ± 7	1.3 ± 0.4	8.5 ± 4.1	0.33 ± 0.18	8.1 ± 3.8	21 ± 8

Position	Field type	Cultivation duration (year)	Ex-Na (cmol_c_ K kg^−1^)	pH	Clay (%)	Silt (%)	Sand (%)	Gravel (%)
Non-cultivation fields (n = 9)		0	0.1 ± 0.0	6.4 ± 0.2	51 ± 7	24 ± 5	16 ± 3	10 ± 4
Slope fields	Upper position (n = 32)	1–10	0.2 ± 0.1	6.7 ± 0.2	41 ± 16	33 ± 11	19 ± 5	7 ± 8
		11–20	0.1 ± 0.1	6.6 ± 0.4	31 ± 11	26 ± 10	27 ± 13	16 ± 22
		21–30	0.2 ± 0.1	6.5 ± 0.5	40 ± 10	25 ± 7	23 ± 10	11 ± 11
	Middle position (n = 32)	1–10	0.2 ± 0.2	6.7 ± 0.2	43 ± 11	29 ± 6	21 ± 7	7 ± 7
		11–20	0.1 ± 0.1	6.6 ± 0.5	33 ± 8	29 ± 7	26 ± 8	12 ± 12
		21–30	0.2 ± 0.1	6.5 ± 0.5	41 ± 11	27 ± 6	24 ± 8	8 ± 8
	Lower position (n = 32)	1–10	0.2 ± 0.2	6.6 ± 0.2	42 ± 15	30 ± 8	21 ± 5	8 ± 6
		11–20	0.1 ± 0.1	6.6 ± 0.5	39 ± 8	32 ± 7	24 ± 10	4 ± 4
		21–30	0.1 ± 0.1	6.5 ± 0.5	45 ± 6	26 ± 5	23 ± 6	6 ± 5
The flat fields	Flat position (n = 24)	1–10	0.1 ± 0.1	6.3 ± 0.0	23 ± 1	25 ± 0	51 ± 2	1 ± 1
		11–20	0.2 ± 0.2	6.7 ± 0.3	36 ± 9	30 ± 10	28 ± 3	7 ± 5
		21–30	0.2 ± 0.1	6.4 ± 0.2	36 ± 12	20 ± 5	29 ± 9	11 ± 9

The values of soil properties are shown as the average value ± standard deviation.

### Relationships between soil and continuous maize cultivation

The results of ANCOVA for analyzing the effect of continuous maize cultivation on soil properties are shown in Table 2. The effect of the duration of continuous maize cultivation on the content of TC, TN, Ex-K, and Ex-Ca was statistically significant. There was a weak effect of continuous maize cultivation on Av-P content (p value < 0.1). Among the four topo-sequential positions, only the effect of the flat position was significant on TC, TN, Ex-K, and sand. The interaction effect between the duration of continuous cultivation and the flat position on TC, TN, Ex-K, and gravel was also significant. The total coefficient of the cultivation duration and the interaction between the cultivation duration and the flat position was close to zero on TC, TN, and Ex-K. This result indicates that the rate of decrease of these soil properties was quite low in flat positions.

**Table 2:** ANCOVA results to estimate the effects of maize cultivation duration and topo-sequential position on soil properties.

	TC	TN	Av-P	Ex-K	Ex-Mg	Ex-Ca
Cultivation duration [D]	− 0.41**	− 0.025***	− 0.36†	− 0.010**	− 0.05	− 0.42*
**Topo-sequential position**						
Lower	0.00	0.000	0.00	0.000	0.00	0.00
Middle [M]	− 0.13	− 0.005	− 1.87	0.002	− 0.11	0.48
Upper [U]	− 1.52	− 0.133	− 0.80	− 0.111	− 0.63	− 1.64
Flat [F]	− 10.86**	− 0.707**	5.60	− 0.307**	− 2.89	− 9.70†
**Interaction**						
[D] × [M]	− 0.11	− 0.005	0.01	− 0.004	0.04	− 0.02
[D] × [U]	− 0.08	− 0.001	− 0.07	− 0.001	0.06	0.15
[D] × [F]	0.42*	0.026*	− 0.05	0.012 *	0.04	0.29
Intercept	29.6***	2.0***	15***	0.59***	11***	31***
R-square	0.30	0.33	0.17	0.29	0.09	0.13
Adjusted R-square	0.26	0.29	0.11	0.24	0.03	0.08

	Ex-Na	pH	Clay	silt	Sand	Gravel
Cultivation duration [D]	− 0.0033	− 0.009	0.17	− 0.23	0.14	− 0.09
**Topo-sequential position**						
Lower	0.0000	0.000	0.00	0.00	0.00	0.00
Middle [M]	0.0004	0.079	− 1.26	− 3.16	1.74	2.67
Upper [U]	− 0.0196	0.017	− 3.89	0.90	0.89	2.10
Flat [F]	− 0.0746	− 0.106	− 2.12	− 5.02	18.84**	− 11.70†
**Interaction**						
[D] × [M]	0.0009	− 0.002	− 0.09	0.12	− 0.05	0.02
[D] × [U]	0.0025	0.001	− 0.04	− 0.13	− 0.04	0.21
[D] × [F]	0.0054	0.000	− 0.23	0.01	− 0.57†	0.78*
Intercept	0.21***	6.7***	39***	33***	20***	7†
R-square	0.03	0.07	0.05	0.13	0.17	0.11
Adjusted R-square	− 0.03	0.01	− 0.01	0.07	0.11	0.06

^†^, *, **, and ***Represent statistical significance at p < 0.1, 0.05, 0.01, and 0.001, respectively.

**Figure 2:**
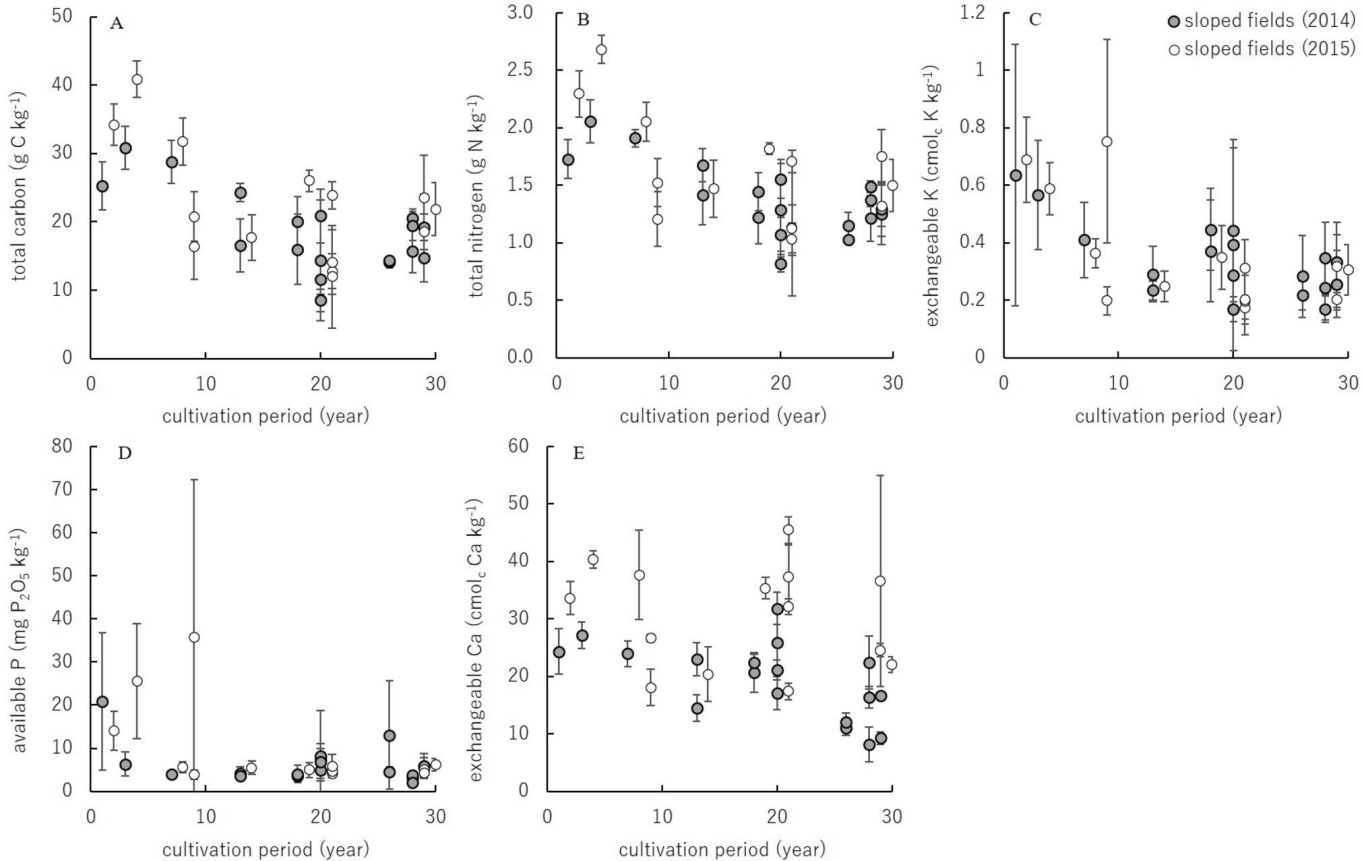
Relationships between the five soil nutrients and the duration of continuous maize cultivation in the sloped fields, **A** totale carbon (TC), **B** total nitrogen (TN), **C** exchangeable potassium (Ex-K), **D** available phosphate (Av-P), and **E** exchangeable calcium (Ex-Ca). Error bars represent the standard deviation of the values from three sample sites of each filed.

Since the interaction of the cultivation duration and topo-sequential position was not significant on TC, TN, Av-P, Ex-K, and Ex-Ca in ANCOVA results in the three topo-sequential positions in the sloped fields, a linear regression analysis was performed for those soil properties in the sloped fields. The R-square values of the regression line for TC, TN, Av-P, Ex-K, and Ex-Ca were 0.30, 0.33, 0.12, 0.24, and 0.11, respectively (Table 3). The decreasing trend of TC, TN, and Ex-K was clear in the sloped fields during continuous cultivation (Fig. 2). Although the relationships between the cultivation duration and the contents of Av-P and Ex-Ca were weak, the effects of the cultivation duration on the soil nutrients were significant (Table 3). Based on the linear regression line, the decreasing rate of TC, TN, Av-P, Ex-K, and Ex-Ca was estimated to be − 0.48 g C kg^-1^ year^-1^, − 0.028 g N kg^-1^ year^-1^, − 0.38 mg P_2_O_5_ kg^-1^ year^-1^, − 0.011 cmol_c_ K kg^-1^ year^-1^, and − 0.37 cmol_c_ Ca kg^-1^ year^-1^, respectively, (Table 3). The initial contents of these soil items were estimated as 29 g C kg^-1^, 2.0 g N kg^-1^, 15 mg P_2_O_5_ kg^-1^, 0.55 cmol_c_ K kg^-1^, and 31 cmol_c_ Ca kg^-1^, respectively. The contents of these soil items in non-cultivation fields were 30 g C kg^-1^, 2.0 g N kg^-1^, 4.1 mg P_2_O_5_ kg^-1^, 0.60 cmol_c_ K kg^-1^, and 21.9 cmol_c_ Ca kg^-1^, respectively. The estimated initial contents of TC, TN, Ex-K, and Ex-Ca were close to the contents observed in non-cultivation fields. However, the estimated initial content of Av-P was four times larger than that in non-cultivation fields. The Av-P content was lower in non-cultivated fields than that in fields with a cultivation duration of less than 10 years.

**Table 3:** Results of the regression analysis to estimate the effect of maize cultivating duration on five soil properties in sloped fields.

	TC		TN		Av-P		Ex-K		Ex-Ca	
cultivating duration	− 0.48***		− 0.028***		− 0.38***		− 0.011***		− 0.37***	
intercept	29.1***		2.0***		15***		0.55***		31***	
R-square	0.30		0.33		0.12		0.24		0.11	
adjusted R-square	0.29		0.32		0.11		0.24		0.10	

***Represents statistical significance at p < 0.001.

### Relationships between soil properties and maize yield

**Figure 3:**
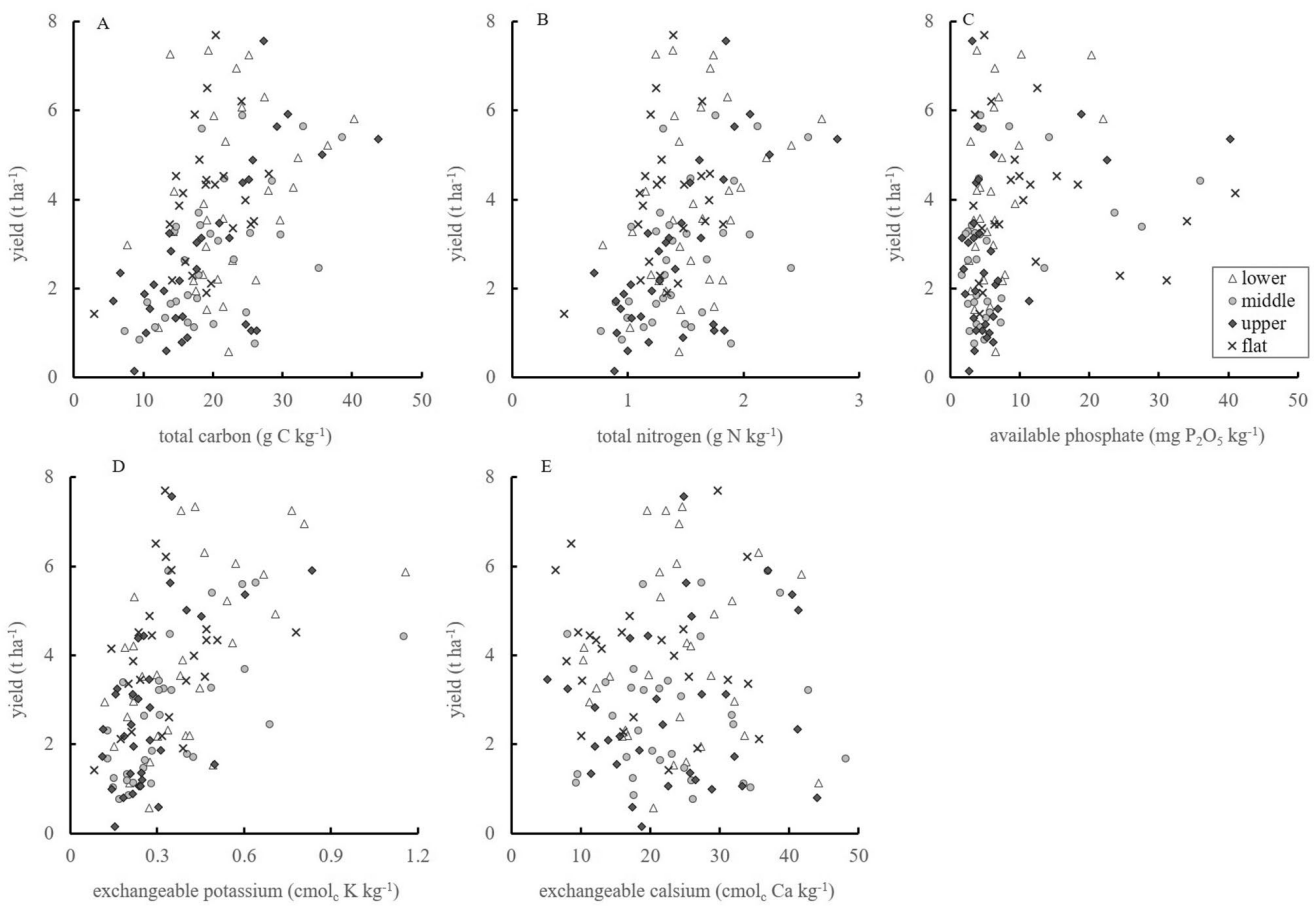
Relationships between the five soil nutrients and maize yield, **A** totale carbon (TC), **B** total nitrogen (TN), **C** exchangeable potassium (Ex-K), **D** available phosphate (Av-P), and **E** exchangeable calcium (Ex-Ca).

Maize yield was weakly related to TC, TN, and Ex-K, and the determination coefficients were 0.24, 0.19, and 0.28, respectively (Fig. 3). The relationship between yield and Av-P was not clear, but yield tended to be high with increasing Av-P. Although Ex-Ca decreased with increasing cultivation duration, yield was not related to Ex-Ca. The results of ANCOVA suggested that the topo-sequential positions and their interactions with soil properties did not have large effects on yield (data not shown).

## Discussion

### Trends in soil properties under continuous maize cultivation

This study evaluated the change in soil properties under continuous maize production using the chronosequence method. The evaluation represents a 30-year change in Kenthao District in Sainyabuli Province, where cultivation management without fertilizer application was the standard practice among farmers^3^. To evaluate the changing trends of soil properties, the current study applied a linear function because the function is generally more robust in these cases. The results of ANCOVA showed that the interactions between the cultivation duration and the three topo-sequential positions in the sloped fields were not significant for TC, TN, Av-P, Ex-K, and Ex-Ca, suggesting that the decreasing rates were not obviously different among the three topo-sequential positions in the sloped fields (Fig. 2). In the sloped fields, soil erosion generally causes nutrient loss, especially in the upper part. Quine and Zhang^20^ reported the presence of relatively higher loss of soil and relatively lower TC content in the upper part of sloped fields. A lower distribution of Av-P in the upper part of the sloped fields was also observed due to soil erosion^21^. In this study, the coefficients of the interaction of the cultivation duration and upper topo-sequential positions were relatively low compared to the lower positions on TC, TN, Av-P, and Ex-K (Table 2), which suggests the possibility of high nutrient loss in the upper part of the sloped fields. However, the results of ANCOVA did not imply the redistribution of soil nutrients by soil erosion in the sloped fields.

The decrease in TC and TN contents might be enhanced by the decomposition of soil organic matter. TC and TN would be decomposed in all topo-sequential positions because soil temperature and moisture are suitable in tropical conditions. However, the decreasing rates of TC and TN were almost zero at the flat positions of the flat fields (Table 1). Further, the contents of TC and TN at all the flat positions were as low as those in the fields where the duration of continuous cultivation was 21 to 30 years in the upper position. This finding indicates that they already reached an equilibrium status of decomposition and input from crop residue. The lower content of TC and TN in the flat fields where the duration of continuous cultivation was 1 to 10 years may be associated with the soil conditions before the reclamation of the forest.

Our previous report showed that maize production has been decreasing during continuous cultivation for 30 years^3^. This phenomenon corresponds to the change in soil properties observed in this study. The contents of TC, TN, Av-P, and Ex-K were associated with maize yield (Fig. 3). This result indicated that the decrease in TC, TN, Av-P, and Ex-K might cause a yield reduction. Numerous studies have also reported the association of soil carbon with soil fertility and consequently, crop productivity^22–24^. The contents of TC, TN, Av-P, and Ex-K correlated with yield, but each soil property explains a small proportion of the yield variance (Fig. 3). It is necessary to analyze the relationship between the soil properties and yield in detail to clarify the mechanism of yield reduction during continuous maize cultivation in the study area.

### Degradation rate of soil properties

Since the estimated initial contents of TC, TN, and Ex-K based on the intercept of the linear regression line were close to the contents observed in non-cultivation fields, the estimated initial contents were presumably close to the actual initial content. In terms of Av-P, unlike other soil nutrients, the estimated initial content was higher than the content in non-cultivated fields. Since the content of Av-P often increased by the reclamation of forest^25,26^, the Av-P content at first maize cultivation might be close to the estimated initial content rather than the content in non-cultivation fields. The decreasing rates and initial contents based on linear regression analysis suggested that TC, TN, Av-P, and Ex-K in surface soil decreased to 50%, 58%, 24%, and 40% of the initial content, respectively, under continuous maize cultivation for 30 years. Our results suggest that soil degradation under continuous maize cultivation has a critical impact in the study area.

The decreasing rate of TC under crop cultivation is generally faster in tropical climate zones than in temperate climate zones because high temperatures accelerate decomposition rate^9^. Moebius-Clune et al.^7^ reported a decreasing rate of organic matter to be around − 2.5 g kg^-1^ year^-1^ in continuous maize cultivation for 40 years in Kenya, from which the decreasing rate of TC is estimated to be − 1.5 g C kg^-1^ year^-1^ according to Guo and Gifford^26^. Sa et al.^27^ reported a decreasing rate of TC − 0.46 g C kg^-1^ year^-1^ in the continuous cultivation of several crops, including maize, for 22 years. In contrast, Dalal and Mayer^11^ found that the decreasing rate of TC was approximately − 0.23 g C kg^-1^ year^-1^ in maize cultivation for 30 years in temperate climate zones such as Australia. In the temperate climate zone of Iran, Shahriari et al.^28^ reported that the decreasing rate of TC ranged from − 0.19 to − 0.28 g C kg^-1^ year^-1^ for 34 years. Similarly, Hajabbasi et al.^29^ reported that the decreasing rate of TC was − 0.77 g C kg^-1^ year^-1^ under wheat and barley cultivation for 20 years in Iran. In the USA (temperate climate zone), Haas et al.^30^ reported that the decreasing rate of TC was − 0.15 g C kg^-1^ year^-1^ under continuous maize cultivation for 30 years. In this study site, the decreasing rate of TC was estimated as − 0.48 g C kg^-1^ year^-1^ in the sloped fields. This decreasing rate was high compared to the rates reported in previous studies in temperate zones. However, the rate of decrease was close to zero in the flat fields (Table 2). The high decreasing rate of the slope field might be caused by soil erosion in addition to high temperature conditions. In flat fields with less impact of soil erosion, TC might decrease slowly even under high temperature conditions. Soil carbon retention is related to clay content^31,32^, clay minerals^33,34^, and climate conditions^35,36^. The relatively high clay content (about 35%) in the study site might mitigate the reduction in the decreasing rate of TC during continuous cultivation. TC content tended to be high in the field with high clay content, which also suggests the role of clay content in retention of TC. The remaining amount of TC per unit clay content varies with clay minerals. Further analysis of clay minerals is necessary to evaluate the decreasing rate of TC more precisely.

In the mountainous areas of Southeast Asia, land use has been changing from forest to crop cultivation. Along this land-use change, the area under maize cultivation has increased. For example, the maize cultivation area has increased more than twice in Indonesia, Vietnam, and Myanmar from 1980 to 2018^37^. Fox and Vogler reported that market pressure was one of the influential factors increasing the cultivation area of cash crops in Southeast Asia^38^. Since maize grain is used for feeding livestock and poultry, the increase in demand of meat in Asia will lead to an increase in the maize cultivation area. In Southeast Asia, because flat fields are mainly used for rice cultivation, maize cultivation will be undertaken in sloped fields. Our results indicate that the decreasing rates of soil nutrients were high in the sloped fields. Valentin et al. reported that maize cultivation in sloped fields was strongly related to soil erosion in five countries in Southeast Asia^39^. Lal reported that soil degradation by erosion caused serious nutrient loss in developing countries^34^. Maize cultivation without appropriate management may decrease soil nutrients by a significant level for several decades in mountainous areas in Southeast Asia. For sustainable food production, management of soil fertility is required in sloped fields under maize cultivation.

## Conclusion

TC, TN, Av-P, Ex-K, and Ex-Ca content in soil showed a decreasing trend in farmers’ maize fields in Sainyabuli province, Laos. These soil properties could have reduced to almost half of the initial content during continuous maize cultivation for 30 years. The rates of decrease of the soil properties were high in the sloped fields. The decrease in TC, TN, Av-P, and Ex-K content is likely to cause a decrease in maize yield. Improvement of soil management is required to retain soil fertility.
